# Genetic and epigenetic changes in the common 1p36 deletion in neuroblastoma tumours

**DOI:** 10.1038/sj.bjc.6604032

**Published:** 2007-10-16

**Authors:** H Carén, S Fransson, K Ejeskär, P Kogner, T Martinsson

**Affiliations:** 1 Department of Clinical Genetics, Institute of Biomedicine, Göteborg University, Sahlgrenska University Hospital, Göteborg SE-41345, Sweden; 2 Childhood Cancer Research Unit, Department of Woman and Child Health, Karolinska Institutet, Karolinska Hospital, Stockholm SE-17176, Sweden

**Keywords:** neuroblastoma, 1p, mutation, epigenetics, *PIK3CD*

## Abstract

Chromosome 1p is frequently deleted in neuroblastoma (NB) tumours. The commonly deleted region has been narrowed down by loss of heterozygosity studies undertaken by different groups. Based on earlier mapping data, we have focused on a region on 1p36 (chr1: 7 765 595–11 019 814) and performed an analysis of 30 genes by exploring features such as epigenetic regulation, that is DNA methylation and histone deacetylation, mutations at the DNA level and mRNA expression. Treatment of NB cell lines with the histone deacetylase inhibitor trichostatin A led to increased gene transcription of four of the 30 genes, *ERRFI1 (MIG-6)*, *PIK3CD*, *RBP7 (CRBPIV)* and *CASZ1*, indicating that these genes could be affected by epigenetic downregulation in NBs. Two patients with nonsynonymous mutations in the *PIK3CD* gene were detected. One patient harboured three variations in the same exon, and p.R188W. The other patient had the variation p.M655I. In addition, synonymous variations and one variation in an intronic sequence were also found. The mRNA expression of this gene is downregulated in unfavourable, compared to favourable, NBs. One nonsynonymous mutation was also identified in the *ERRFI1* gene, p.N343S, and one synonymous. None of the variations above were found in healthy control individuals. In conclusion, of the 30 genes analysed, the *PIK3CD* gene stands out as one of the most interesting for further studies of NB development and progression.

Neuroblastoma (NB) is the most common extracranial tumour of childhood (Gale *et al*, 1982). One of the hallmarks of NB tumours is their clinical heterogeneity, ranging from spontaneous regression to malignant disease. Deletion of the short arm of chromosome 1 (1p-deletion), additional genetic material from the long arm of chromosome 17 (17q gain) and amplification of the proto-oncogene *MYCN* are examples of chromosomal abnormalities that have been found in NB. The 1p region has been subjected to intense study in this tumour type; it shows loss of heterozygosity (LOH) in 20–40% of NB tumours. 1p-deletion is also highly correlated with *MYCN* amplification and predicts unfavourable outcome (Caron *et al*, 1996). It has therefore been proposed that the region contains a tumour suppressor gene that is inactivated in aggressive NB tumours.

The deletion of chromosome 1 often involves a large proportion of 1p but some tumours display smaller deletions. Our group, as well as others, has tried to identify the critical region/regions by comparing the deletions found in the tumours. We have defined the shortest region of overlap (SRO) of deletions in our tumour material to about 25 cM located between the markers D1S80 and D1S244 (Martinsson *et al*, 1995, 1997). By the addition of germ cell tumours, an approximately 5 cM combined SRO of deletions was defined by markers D1S508 and D1S244 (Ejeskär *et al*, 2001). As an overlapping homozygous 500 kb deletion of 1p36.2–3 was found in an NB cell line (Ohira *et al*, 2000), the region has been analysed in further detail. In our study of the genes within this region, all seven were screened for mutations and a few were indeed discovered (Ejeskär *et al*, 2000; Abel *et al*, 2002, 2004; Krona *et al*, 2003, 2004). We have also explored the expression and methylation status of these genes. The transcripts have been shown to be downregulated in unfavourable NB, compared to favourable NB, a feature that cannot be explained by methylation of their respective CpG islands (Carén *et al*, 2005). In the current study, we wanted to expand our investigation of the 1p region, more specifically to our combined NB/germ cell SRO of deletions, and to explore epigenetic mechanisms in the regulation of possible tumour suppressor genes. The effect of DNA methylation and histone deacetylation events of 30 genes in the 1p36 chromosomal region extending from markers D1S508 and D1S244, bp 7 765 595–11 019 814 (UCSC version, May 2004; URL: http://genome.ucsc.edu) were explored. This SRO of deletions is in agreement with SRO studies presented by other groups (Caron *et al*, 2001; Chen *et al*, 2001). Yet, other groups have presented SRO located more distal on 1p (Bauer *et al*, 2001; White *et al*, 2005).

Genes identified as possibly regulated by epigenetic means were studied further with expression analysis and mutation screening of primary tumours. A small number of the 1p genes studied showed indication of epigenetic inactivation and two of these also contained mutations in NB tumours.

## MATERIALS AND METHODS

### Cell lines and patients

A panel of 66 primary NB tumours of different stages was used in the study, 35 tumours were used for expression analysis; 17 tumours with favourable biology from patients with no evidence of disease and 18 tumours with unfavourable biology from patients who have died from the disease and 46 for DNA sequencing (Table 1). Fifteen of the samples were used for both expression and sequencing analysis. Also, 120 healthy control individuals were used for DNA sequencing. For cell treatments, three NB cell lines with 1p-deletion (IMR-32, SK-N-AS, SK-N-BE(2)) and one with intact 1p (SH-SY5Y) were used. These and five other NB cell lines (SK-N-DZ, SK-N-F1, SK-N-SH, Kelly and NB69) were used for bisulphite sequencing.

### Analysis of methylation and acetylation status

Cells were seeded at low density and treated with the demethylating agent 5-Aza-2′-deoxycytidine (5-Aza-dC) (Sigma-Aldrich Co, St Louis, MO, USA) or with the histone deacetylase inhibitor trichostatin A (TSA; Sigma-Aldrich) on the day after seeding. Different concentrations and exposure durations were investigated and a concentration of 2 or 4 *μ*M of 5-Aza-dC for 72 h and 0.5 *μ*M of TSA for 16 h were chosen. The experiments were repeated twice and medium was changed every second day to fresh medium containing the respective agents. As controls, the respective cell lines were mock treated with the same amount of carrier (EtOH for the TSA treatments and DPBS; Dulbecco's buffered saline, PAA Laboratories, Linz, Austria, for 5-Aza-dC).

### Expression analysis

#### cDNA preparation

Total RNA was extracted from the cell lines using the RNeasy RNA extraction kit (Qiagen, Hilden, Germany). Total RNA from NB tumour samples was extracted using the RNA extraction kit or Totally RNA (Ambion, Austin, TX, USA). Total RNA, 1 *μ*g, was reverse transcribed to cDNA using Superscript II (Amersham, Buckinghamshire, UK) and random hexamer primers, all according to the protocol of the supplier. The cDNA samples were quality-tested by amplification of the *GUSB* (*β*-glucuronidase) gene.

#### Real-time RT–PCR – endogenous control

The *GUSB* gene was used as an endogenous control for normalisation of expression in the tumour samples. This gene has previously been shown to be expressed at constant levels in tumour samples, regardless of NB stage (Abel *et al*, 2005). In order to select the most appropriate endogenous control for the NB cell lines, untreated and treated cell lines were tested for their expression levels of seven commonly used housekeeping genes using TaqMan® Assays-on-Demand™ Gene Expression Products (Applied Biosystems, Foster City, CA, USA). Analysis was performed with geNorm 3.4 software (Vandesompele *et al*, 2002) which determines the most stable housekeeping genes in a set of genes in the cDNA panel. *GUSB*, *UBC* (*β*_2_-microglobulin) and *SDHA* (succinate dehydrogenase) showed the smallest variations in Δ*C*_T_ levels and were expressed at constant levels in samples regardless of treatment; these genes were therefore used as internal references for normalisation in the real-time RT–PCR quantification analysis for the NB cell lines.

#### Real-time RT–PCR – TaqMan

TaqMan primers and probes were derived from Applied Biosystems. Real-time RT–PCR was performed in 384-well plates using the ABI PRISM® 7900HT Sequence Detection System (Applied Biosystems). Amplification reactions (10 *μ*l) were carried out in duplicate with 0.1 *μ*l template cDNA, according to the protocol of the manufacturer (Applied Biosystems). A standard curve with six cDNA dilutions was recorded and two nontemplate controls were included in each assay.

Quantification was performed by the standard curve method, as described previously (Abel *et al*, 2005). Briefly, the mean *C*_T_-value for duplicates was calculated, and the gene concentration (or gene copy numbers) of test samples was interpolated based on standard curves. All samples were normalised by dividing the concentration of the test gene with the concentration of the housekeeping gene/genes in the same cDNA sample.

The logarithms of the expression levels in favourable and unfavourable NB tumours were compared using Student's two-sided *t*-test. Box plots were constructed using SPSS 12.0.1 for Windows.

### Confirmation of methylation status

#### Bisulphite modification

DNA was phenol-extracted using phase lock gel (Eppendorf AG, Hamburg, Germany) according to standard procedure and was, with some minor changes, modified with bisulphite according to previously published papers (Clark *et al*, 1994; Paulin *et al*, 1998). Briefly, 1 *μ*g of genomic DNA was treated with restriction endonucleases that digested the DNA close to, but outside, the region of interest. The DNA was then denatured in 0.3 M freshly prepared NaOH at 40°C for 15 min. Sodium metabisulphite (Sigma-Aldrich) and urea, at final concentrations of 1.73 M and 5.36 M, respectively, were added in order to sulphonate the unmethylated cytosines, together with hydroquinone (0.5 mM). Conversion was carried out at 55°C for 16 h, with a temperature increase to 95°C for 30 s every 3 h. DNA was purified with the Wizard DNA cleanup system (Promega Corporation, Madison, WI, USA), according to the instructions of the manufacturer, and desulphonated in 0.3 M NaOH at 37°C for 15 min and finally precipitated in ethanol, resuspended in distilled H_2_O and stored at −20°C. Universally Methylated DNA (Chemicon International, Temecula, CA, USA) was included as a positive control for methylation.

#### Promoter analysis and DNA amplification

Prediction of promoters associated with CpG islands were done with CpGProD (Ponger and Mouchiroud, 2002) and CpG islands were also searched with CpG island searcher (URL: http://cpgislands.usc.edu). Criteria for CpG island selection were chosen according to Takai and Jones (2002), that is an expected GC content of >55%, an observed/expected CpG ratio of >0.65 and >500 bp. The regions were also searched with relaxed criteria's. The regions, or parts of them, were amplified with one primer pair or, if required, with nested primers (primer sequences available on request). The methylation status was analysed using bisulphite sequencing. Touchdown PCR was performed with 1 × Reaction Buffer, 0.5 mM dNTPs, 2.0–3.0 mM MgCl_2_, 0.4 *μ*M of forward and reverse primers, respectively, and 1 U of HotStar *Taq* (Qiagen, Hilden, Germany), in a total volume of 20 *μ*l. Reactions were denatured at 95°C for 15 min, followed by five cycles of 95°C for 1 min, 5°C above annealing temperature with a decrease of 1° per cycle for 1 min, 72°C for 1 min and 30 cycles of 95°C for 1 min, annealing temperature for 1 min, 72°C for 1 min and ending with 7 min extension at 72°C. PCR products were purified with ExoSAP-IT^TM^ (USB Corporation, Cleveland, OH, USA) and sequencing was carried out using forward or reverse primer with the ABI Prism BigDye^TM^ cycle sequencing Ready Reaction Kit (Applied Biosystems). The samples were analysed in an ABI 3100 Genetic Analyzer or an ABI 3730 Genetic Analyzer (Applied Biosystems). Sequence analysis was conducted with SeqScape version 2.1.1 (Applied Biosystems).

### DNA mutation screening

#### DNA amplification

Primers were designed for the exons and flanking intronic sequences using the Exonprimer feature of the UCSC genome browser (URL: http://genome.ucsc.edu/) and were ordered from Life Technologies, Inc., Gaitherburg, MD, USA (primer sequences available on request). Standard reactions of 20 *μ*l were used, containing 25–100 ng DNA, 1.5 mM MgCl_2_, 2 mM dNTP, 0.6–0.75 *μ*M primer and 1 U *Taq* polymerase (Amersham Pharmacia Biotech, Freiburg, Germany). Reactions were denatured at 95°C for 2 min, followed by 35 cycles of 95°C for 30 s, annealing for 30 s, 72°C for 1 min, and ending with a 7 min extension step. Purification of PCR reactions and sequencing were performed as described above.

## RESULTS

### Expression analysis of cells treated with TSA and 5-Aza-dC

*GUSB*, *UBC* and *SDHA* were selected as endogenous controls for real-time RT–PCR quantification and used as internal references for normalisation. Four of the genes in the study, *ERRFI1 (MIG-6)*, *PIK3CD*, *RBP7 (CRBPIV)* and *CASZ1*, were upregulated more than two-fold after treatment with TSA (Table 2) in both experiments. Expression of gene transcripts of these four genes was analysed in primary NB tumours and the DNA sequences were analysed for mutations. Three genes, *PIK3CD*, *RBP7* and *CASZ1*, were upregulated in at least two of the cell lines after treatment with 5-Aza-dC. These genes were analysed further with bisulphite sequencing.

### Bisulphite sequencing

**Figure 1:**
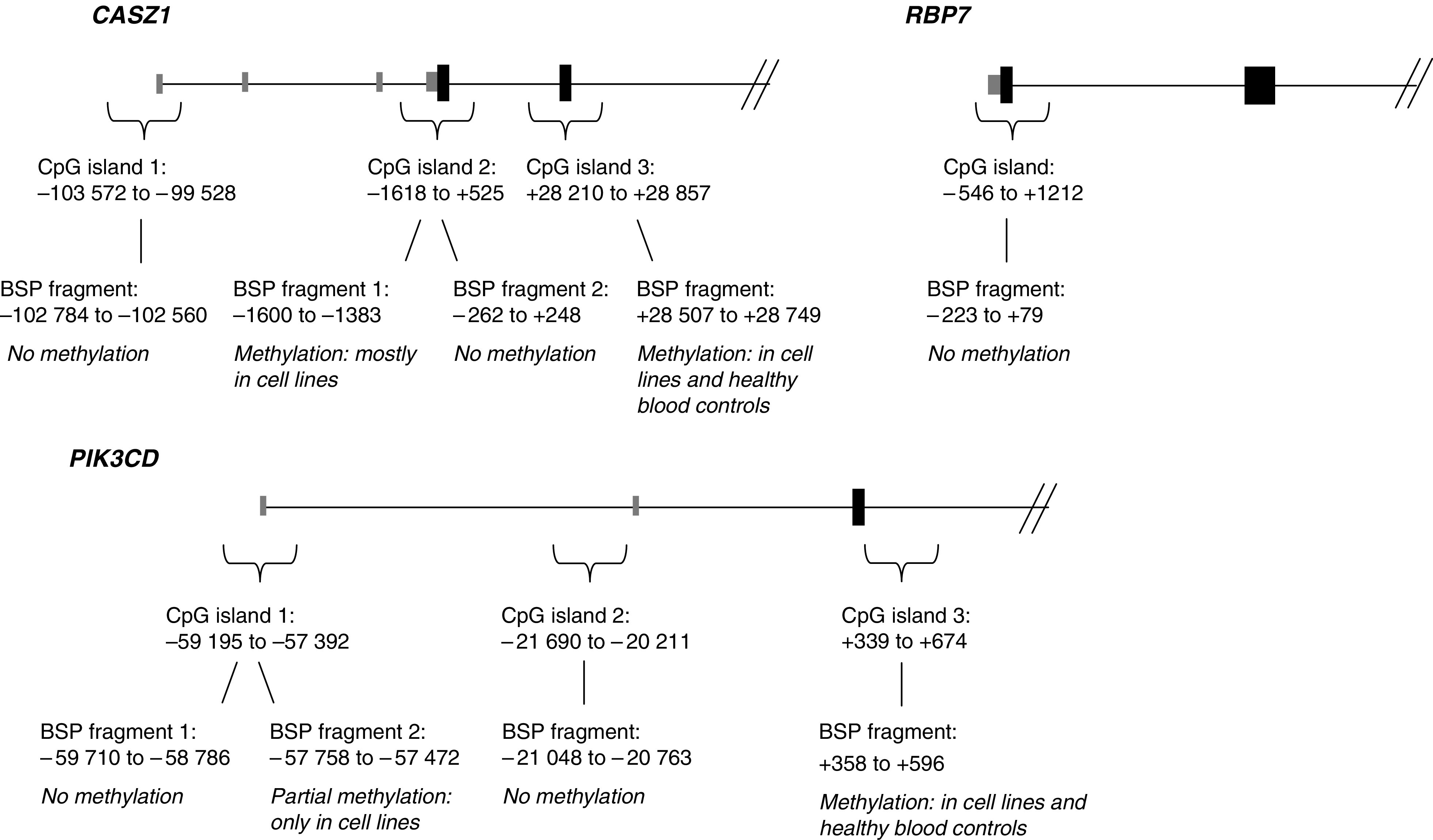
The *CASZ1, RBP7* and *PIK3CD* genes. Black boxes indicate coding exons and grey boxes untranslated exons. Positions with the A in the initiator Met codon denoted nucleotide +1. CpG islands number 3 in *CASZ1* and *PIK3CD* were identified with relaxed searching criteria (an expected GC content of >50%, an observed/expected CpG ratio of >0.6 and >200 bp).

**Figure 2:**
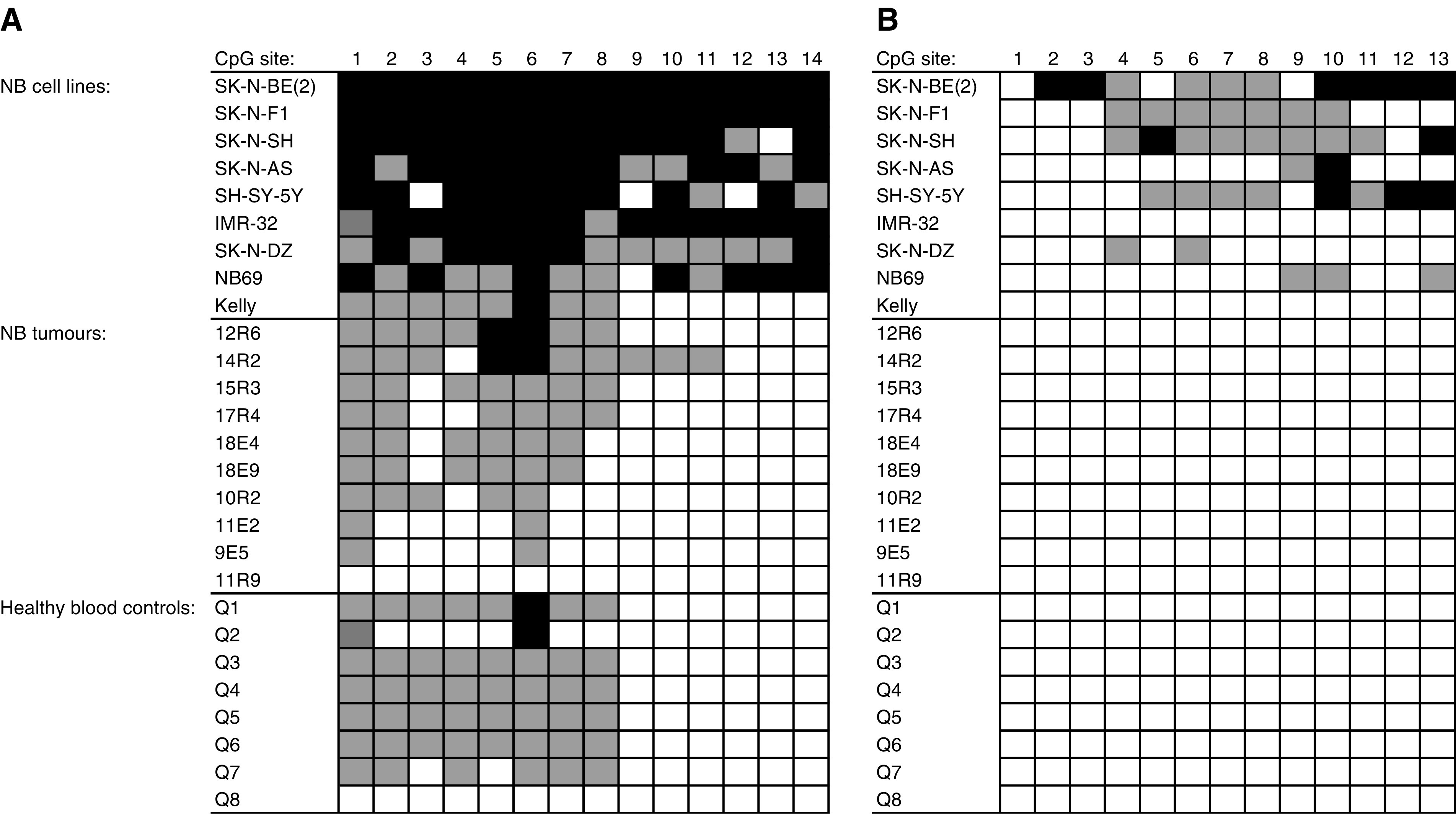
Methylation status of (**A**) *CASZ1* CpG island 2 fragment 1 and (**B**) *PIK3CD* CpG island 1 fragment 2. Black boxes indicate methylation, grey boxes partial methylation and white boxes no methylation.

*CASZ1*, *PIK3CD* and *RBP7* were studied with bisulphite sequencing. Three CpG islands were studied in *CASZ1* and *PIK3CD.* One or two fragments in each island were PCR amplified and sequenced following bisulphite modification. For location of CpG islands relative to the respective gene, see Figure 1. In our material, NB cell lines generally were found to have more methylated CpG sites than primary NB tumours (Figure 2). No consistent CpG methylation sites distinguishing DNA from primary tumours from that of healthy blood control DNA could be identified. The fragment analysed in the CpG island of *RBP7* was unmethylated in all cell lines.

### Expression analysis of NB tumours

**Figure 3:**
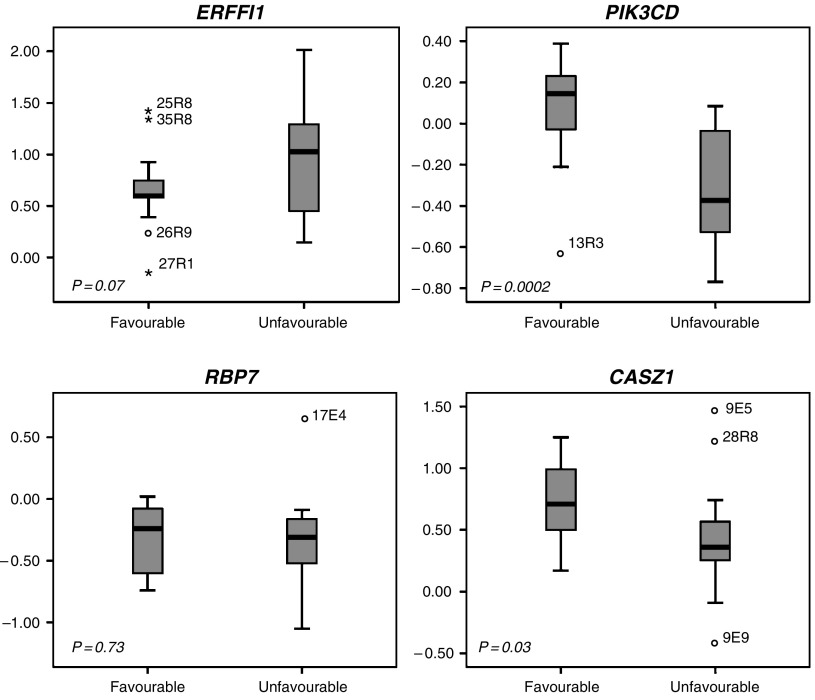
Relative expression of tumours with favourable biology compared to tumours of unfavourable biology. Box plot explanation; upper and lower hinge of the box represent 75th percentile and 25th percentile, respectively; whiskers indicates range; thick horizontal line within box, median. Open circles represent outliers and asterisks represent extremes. The *P*-value at gene-by-gene level is indicated in lower left corner in each graph.

Expression analysis of *ERRFI1, PIK3CD, RBP7* and *CASZ1* was performed comparing 17 tumours with favourable biology from patients with no evidence of disease and 18 tumours with unfavourable biology (dead of disease). The expression of *PIK3CD* was significantly lower (*P*=0.001 after Bonferroni correction), in unfavourable tumours as compared to favourable NB (*PIK3CD*: fold change (fc)=−2.5, *P*=0.0002; *CASZ1*: fc=−2.0, *P*=0.03). No significant difference in expression of *ERRFI1* (fc=+2.1, *P*=0.07) or *RBP7* (fc=−1.1, *P*=0.73) between favourable and unfavourable tumours could be shown (Figure 3).

### DNA sequencing

**Figure 4:**
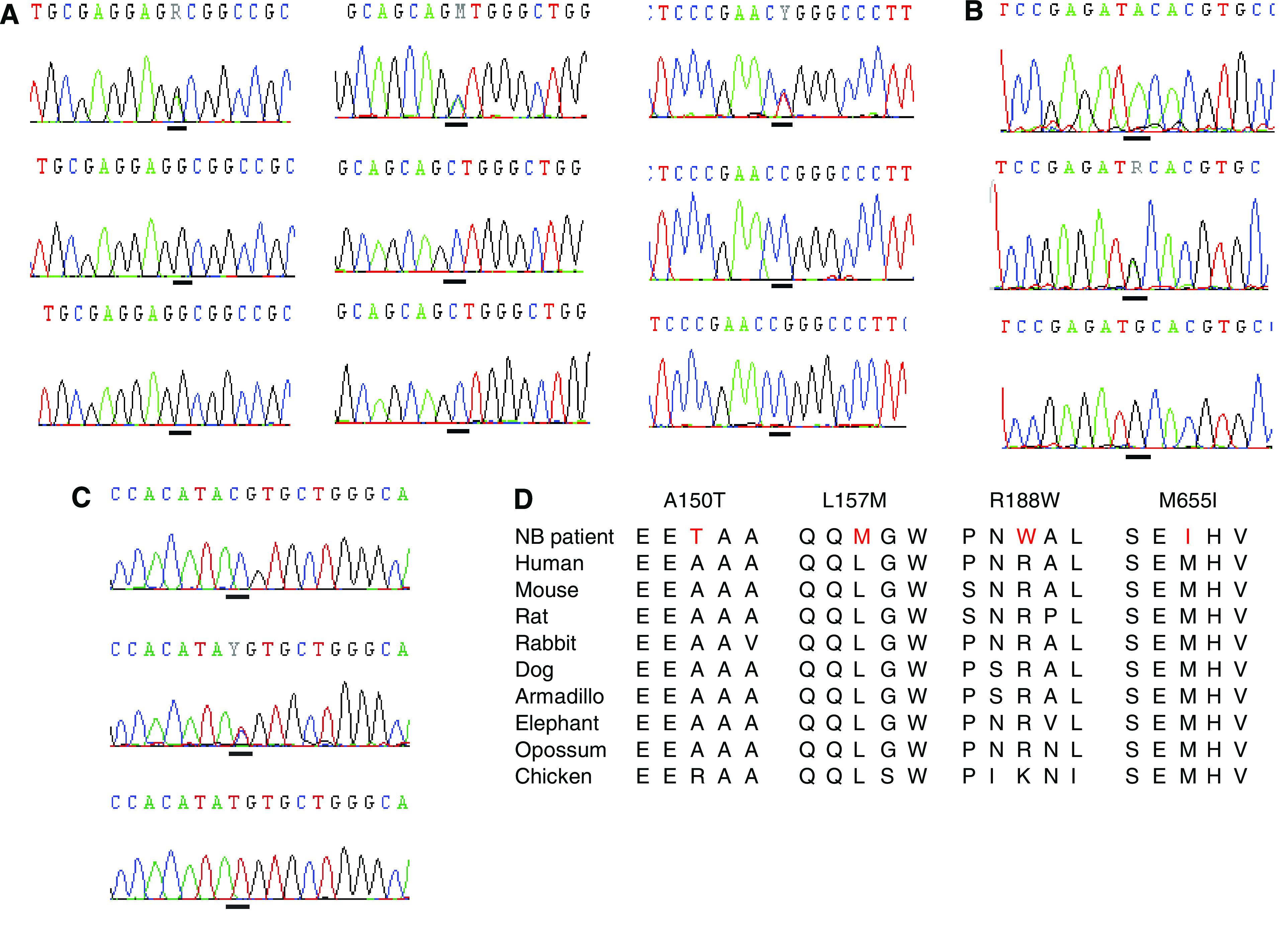
*PIK3CD* mutations in NB primary tumours. Bars under each chromatogram indicate the mutation position. (**A**) Variations in exon 5. Upper panel: Mutations 448G>A, 469C>A and 562C>T in patient 24R3 gave rise to amino-acid changes from Ala to Thr, Leu to Met and Arg to Trp, respectively. Middle panel: Normal tissue from patient 24R3. Lower panel: Healthy control individuals. (**B**) Variation in exon 16. Upper panel: 1965G>A mutation results in amino-acid change from Met to Ile in patient 19R6. Middle panel: Normal tissue from patient 19R6, heterozygous for G/A. Lower panel: Healthy control individual. (**C**) Variation in exon 21. Upper panel: 2661T>C mutation in patient 19R6. Middle panel: Normal tissue from patient 19R6, heterozygous for T/C. Lower panel: Healthy control individual. (**D**) Alignment of amino-acid sequences. The putative mutations, marked with red, are located in conserved regions.

**Figure 5:**
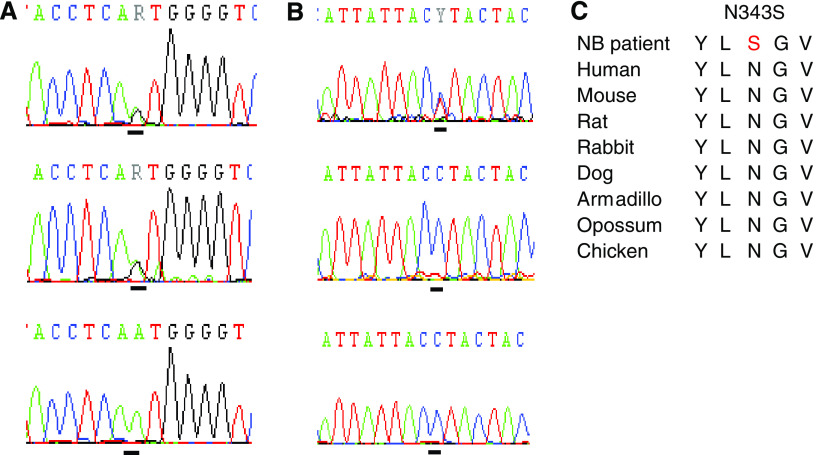
*ERRFI1* in exon 4 in NB tumours. Bars under each chromatogram indicate the mutation position. (**A**) Upper panel: The 1028A>G mutation in patient 25R9 leads to an amino-acid substitution from Asn to Ser. Middle panel: Normal tissue from patient 25R9, heterozygous for A/G. Lower panel: Healthy control individual. (**B**) Upper panel: 1186C>A variation in patient 24R3. Middle panel: Normal tissue from patient 24R3, homozygous for the C allele. Lower panel: Healthy control individual. (**C**) Alignment of amino-acid sequences. The putative mutation, marked with red, is located in a highly conserved region.

Several sequence variations were identified in *ERRFI1* and *PIK3CD* (see Table 3 for a summary). Three patients harboured mutations with amino-acid changes in the *ERRFI1* and *PIK3CD* genes. In exon 5 in *PIK3CD*, three changes were found in the same tumour, 24R3 (see Figure 4). The change, 448G>A, give rise to an amino-acid substitution from the nonpolar amino-acid alanine to the polar threonine, the 469C>A substitution from leucine to methionine (both nonpolar) and 562C>T leads to an amino-acid substitution from the polar arginine to the nonpolar tryptophan. The changes are *de novo* mutations, not present in constitutional DNA from the tumour. In exon 16 in *PIK3CD*, methionine (codon ATG) is changed to isoleucine (codon ATA) in tumour 19R6. The tumour is hemizygous for the variation as the other allele is deleted in the tumour; normal tissue from the patient is heterozygous for the variation. In *ERRFI1*, an amino-acid change from aspartic acid to serine, p.N343S, was found in exon 4, 1028A>G (see Figure 5). This variation was also found in the constitutional DNA from the same patient (25R9). Also, synonymous base changes were identified in *PIK3CD* and *ERRFI1*, see Table 3. None of the alterations described above could be detected in any of 100 healthy control individuals (>200 alleles). In addition to these tumour-specific variations, some novel polymorphisms were identified (Table 3).

## DISCUSSION

1p-deletion is common both in NB and in other tumour types. Since methylation and other epigenetic features have been shown to be important mechanisms in the downregulation and repression of genes, we decided to study DNA methylation and histone deacetylation of genes in the NB/germ cell SRO we had previously defined in order to pinpoint specific genes with a possible involvement in NB. A number of NB cell lines were thus treated with the demethylating agent 5-Aza-dC or the deacetylase inhibitor TSA and the expression of a number of chromosome 1p36.1–2 genes were studied with and without treatment. The genes found to be upregulated after treatment of the NB cell lines were consequently considered to be tentative targets of epigenetic events in NB tumour initiation/progression. The genes thus identified were subjected to (i) bisulphite sequencing of the CpG islands, (ii) analysis of expression in a large number of primary tumours and (iii) mutation screening in the coding regions.

Expression of the *ERRFI1*, *PIK3CD*, *RBP7* and *CASZ1* genes increased after treatment with the deacetylase inhibitor TSA, suggesting that these genes are regulated by histone modifications in NB. *PIK3CD*, *RBP7* and *CASZ1* also exhibited changes in expression in some of the cell lines tested after treatment with the demethylating agent 5-Aza-dC, indicating that these genes could also be silenced by DNA methylation.

The genes we identified as potential targets of epigenetic modification were sequenced using tumour DNA modified with the bisulphite method in order to explore methylation status. The gene sequences were studied with the CpG island searcher and CpGProD (Figure 1). *ERRFI1 (MIG-6)* was not subject to this analysis since its expression was not upregulated after treatment with 5-Aza-dC; furthermore, it displayed a higher level of expression in patients with unfavourable outcome than in patients with favourable outcome.

In our data, there were generally more methylated CpG sites in NB cell lines than in primary NB tumours. This pattern has also been seen in other studies, for example of *CASP8* and *RASSF1A* (Lazcoz *et al*, 2006). No consistent CpG methylation sites in both NB cell lines and primary tumours differing from those of control blood DNA could be identified. Since NB is derived from neural crest progenitor cells, blood DNA might not be a good control. One could speculate that the normal progenitor cells should be completely unmethylated while the cells that develop into NB are methylated, but this is only a speculation. However, since only portions of one of the CpG islands in *CASZ1* and *PIK3CD* (Figure 1) were methylated, it is not likely that this account for the low expression of the genes in unfavourable tumours. The increase in expression after treatment with 5-Aza-dC could be due to the methylation seen in these fragments in the cell lines or alternatively be explained by other normally methylated genes being activated as a result of the treatment which could have an enhancing effect on the transcription of *PIK3CD* and *CASZ1*. Enhancers or other regulatory sequences located outside the analysed region could also be affected by methylation. Other means of silencing could also be involved, as histone modifications, since treatment with TSA increases expression in the NB cell lines. The fragment analysed in the CpG island of *RBP7* was unmethylated in the cell lines used (data not shown, available on request).

The RNA expression analysis showed a decrease in *PIK3CD* and *CASZ1* in aggressive NB, compared to more favourable NB tumours, for *PIK3CD* this decrease was significant also after Bonferroni correction (*P*=0.001). These data are concordant with a previous study of expression in NB of 30 genes in the 1p36.2 region from our group (Fransson *et al*, 2007). The NB tumour material used in that study is overlapping with this previous study; however in this study, we have used clinical outcome criteria for grouping the tumours. One could speculate that the difference in expression could be due to a dosage effect since a major proportion of the unfavourable NB tumours harbours a deletion of 1p.

The RNA expression analysis of *ERRFI1* showed a two-fold increase in unfavourable tumour compared to favourable, seemingly contradicting the results of another study that found *ERRFI1* downregulation in breast tumours in patients with poor prognosis (Amatschek *et al*, 2004). This may reflect different functions, depending on tumour type, but it could also indicate that more advanced stage tumours grow more rapidly since *ERRFI1* expression can be induced by a variety of stimuli such as growth factors, hypoxia and stress factors (Saarikoski *et al*, 2002; Pante *et al*, 2005).

We also performed mutation screening of all coding regions of the four genes, *ERRFI1*, *PIK3CD*, *RBP7* and *CASZ1*, by DNA sequencing. Three tumours with amino-acid changes were identified. Tumour 24R3 has three nonsynonymous mutations in the gene *PIK3CD*. The tumour harbours 1p-deletion, but has two alleles at the site of the mutations according to DNA sequencing (see Figure 4), hence the deletion does not cover this region or more probably, the wild-type allele comes from contaminating normal cells in the DNA sample, indicated by single-nucleotide polymorphisms (SNP) array analysis carried out on the tumour (data not shown). The mutations are *de novo* mutations since they are not found in the constitutional DNA. Tumour 19R6 also harbours a nonsynonymous DNA mutation, M655I. The tumour is 1p-deleted and the normal tissue from the patient is heterozygous for the base variation. A nonsynonymous DNA mutation was also identified in tumour 25R9 (intact 1p) in the *ERRFI1* gene. The variation is also found in constitutional DNA from the patient. Also, one synonymous base change was found in *ERRFI1* and two in *PIK3CD* (one located in intronic sequence). None of the variations mentioned above were detected in any of more than 100 healthy control individuals (more than 200 alleles), indicating that these changes are indeed mutations. Although, it should be noted that samples with mutations are limited. The tumour from patient 25R9 have intact chromosome 1p, so the mutation only constitute one ‘hit’. The mutation in tumour 19R6 in the *PIK3CD* fits the two-hit hypothesis of tumour suppressor inactivation (Knudson, 1971) since it is also 1p deleted. This is probably also true for 24R3 if assuming that the wild-type allele comes from contaminating normal material as indicated by the SNP array analysis of this tumour. Some alterations not annotated as single-nucleotide polymorphisms (SNP) in the UCSC genome browser were also found in the study, indicating that rare polymorphisms have been detected.

The known functions of the four genes are interesting in relation to NB. *ERRFI1*, also known as *MIG-6* or *RALT*, can be induced by stress, growth factors and the protein Ras. *ERRFI1* has recently been reported by Zhang *et al* (2007) to be mutated in human non-small-cell lung cancer cell lines and in one primary tumour. Loss of activity contributes to the initiation of lung carcinogenesis and also other tumour types. RBP7 is a cellular retinoid-binding protein. Retinol is important in embryonic development. *RBP7* is epigenetically silenced by DNA methylation in the promoter region in a high frequency of nasopharyngeal carcinomas as well as in some cancer cell lines (colon, prostatic and ovarian cancer) (Kwong *et al*, 2005). *CASZ1* is a putative homologue to the zinc-finger transcription factor Castor, required for CNS neuronal development in *Drosophila melanogaster*, where it is involved in neuronal cell lineage specification (Mellerick *et al*, 1992; Edenfeld *et al*, 2002). Liu *et al* (2006) has recently presented cloning and characterisation of the human homologue. They reported that the expression of *CASZ1* is increased when cells of neural origin are induced to differentiation. *PIK3CD*, encoding the protein p110*δ*, is a catalytic subunit in class IA phosphoinositide 3-kinase (PI3K). Phosphoinositide 3-kinase are important in regulating signalling involved in cell cycle progression, cell growth, survival and migration. Class 1 PI3K encompasses four isoforms, besides p110*δ*, also p110*α* (*PIK3CA*), p110*β* (*PIK3CB*) and p110*γ* (*PIK3CG*). The four isoforms are believed to have distinct functions and are also regulated differently (Chang *et al*, 1997). Gain of function of the *α* subunit is common in human cancers by overexpression or mutations (Samuels and Velculescu, 2004; Samuels *et al*, 2004). In our NB tumour material, we could not identify any mutations in the *PIK3CA* gene (data not shown) and Dam *et al* (2006) have reported only infrequent mutations in their NB material. The PI3 kinases are generally considered to function as oncogenes. Although, our data could not find any indications of *PIK3CD* acting as an oncogene in NB based on the following aspects: (a) *PIK3CD* is located in a chromosomal region where LOH is common in NB as well as in other paediatric tumours (Grundy *et al*, 1994; Benn *et al*, 2000; Bridge *et al*, 2000), (b) gene expression studies show a downregulation of transcripts in high-stage NB compared to low-stage (consistent with findings of an expression profiling of selected genes of chromosome region 1p35–36 reported by Janoueix-Lerosey *et al*, 2004), (c) 5-Aza-dC and TSA studies indicate that *PIK3CD* could be influenced by epigenetic regulation in NB, (d) putative mutations have been identified. One could speculate that the mutations identified are gain-of-function mutations that would support the concept that the *PIK3CD* gene could act as an oncogene in NB. The downregulation in gene transcripts seen in high-stage compared to low-stage tumours could be an upregulation in both high- and low-stage NB compared to the transcription in the cells from which the NB tumour cells arise. Although, expression analysis of the tumours that harbour the mutations show that the gene is low expressed (data not shown). This contradicts the concept of the mutations being gain-of-function mutations. Further studies to evaluate the function of the *PIK3CD* gene in NB are ongoing.

In summary, we have undertaken a broad analysis of the region located in our NB/germ cell SRO of deletions. Epigenetic regulation, mRNA expression and mutation screening at the DNA level were explored. A group of genes have been identified as epigenetically affected in NB cell lines; the *PIK3CD* gene stands out as the most intriguing, since it also carries mutations in primary tumours, two patients with nonsynonymous mutations were identified. The mRNA expression of this gene is downregulated in unfavourable, compared to favourable, NB tumours. Treatment of NB cell lines with the histone deacetylase inhibitor TSA led to increased gene transcription, indicating that the gene could be epigenetically regulated. DNA mutations were also identified in the *ERRFI1* gene. The current study further strengthens the concept of chromosome region 1p36 being important in the development of NB tumours and supports the hypothesis that there could be several genes in the region required for the initiation and/or progression of this tumour.

## Figures and Tables

**Table 1:** Clinical data of primary neuroblastoma used in the study

**Patient**	**Stage**	**Outcome**	**1p-del**	**MYCN amplification**	**Group in expression analysis**
35R8	1	NED	Neg	Neg	F
14E6	1	NED	Neg	Neg	F
16E1	1	NED	Neg	Neg	F
18E5	1	NED	Neg	?	F
10R7	1	NED	Neg	Neg	F
26R9	1	NED	Neg	Neg	F
9R2	1	NED	Neg	Neg	F
30R9	1	NED	Neg	Neg	F
25R7	1	NED	Neg	Neg	
19R6	1	DOD	Pos	Pos	
25R9	2	NED	Neg	Neg	F
20R9	2	NED	Neg	Neg	F
23R4	2	NED	Neg	Neg	F
25R8	2	NED	Neg	Neg	F
35R2	2	NED	Neg	Neg	F
35R3	2	NED	Neg	Neg	F
13R3	2A	NED	Neg	Neg	F
27R1	2A	NED	Neg	Neg	F
14R9	2B	NED	Pos	Neg	
33R7	2B	NED	Neg	Neg	F
12R4	3	NED	Pos	Neg	
15R8	3	NED	Pos	Neg	
16R4	3	NED	Neg	Pos	
20R8	3	NED	Pos	Pos	
23R2	3	NED	Pos	Pos	
30R7	3	?	Pos	Pos	
13E5	3	DOD	Neg	Pos	
13E6	3	DOD	Pos	Pos	
6E9	3	DOD	Neg	Neg	
10R8	3	DOD	Pos	Neg	
13R1	3	DOD	Pos	Pos	UF
9R9	3	DOD	Pos	Neg	UF
10E6	4	NED	Pos	Pos	
23R5	4	NED	Neg	Neg	
23R8	4	NED	Pos	Pos	
24R3	4	NED	Pos	Pos	
33R9	4	NED	Neg	Neg	
11R6	4	NED	Pos	Pos	
12R9	4	NED	Pos	Pos	
29R2	4	NED	Pos	Pos	
32R2	4	NED	Pos	Neg	
27R4	4	DOD	Neg	Neg	UF
18E4	4	DOD	Pos	Pos	UF
10R2	4	DOD	Pos	Pos	UF
13R0	4	DOD	Pos	Pos	UF
15R3	4	DOD	Pos	Neg	UF
26R8	4	DOD	Pos	Pos	UF
28R8	4	DOD	Neg	Neg	UF
10E7	4	DOD	Neg	Neg	UF
12E6	4	DOD	Neg	Pos	UF
15E5	4	DOD	Pos	Neg	UF
16E3	4	DOD	Pos	Pos	UF
17E4	4	DOD	Pos	Pos	UF
4E1	4	DOD	Neg	Neg	UF
17R2	4	DOD	Neg	Neg	UF
11E2	4	DOD	Neg	Neg	UF
9E5	4	DOD	Pos	Pos	UF
18E9	4	DOD	Pos	Pos	
11R9	4	DOD	Pos	Neg	
12R6	4	DOD	Pos	Pos	
17R4	4	DOD	Pos	Pos	
19R0	4	DOD	Pos	Neg	
21R0	4	DOD	Pos	Pos	
23R7	4	DOD	Pos	Pos	
34R0	4	DOD	Neg	Neg	
12E3	4	DOD	Pos	Pos	
11E5	4S	DOD	Neg	Neg	
14R2	4S	DOD	Pos	Pos	

NED=no evidence of disease; DOD=dead of disease; 1p-del=1p-deletion; Pos=positive; Neg=negative; F=favourable; UF=unfavourable.

**Table 2:** Analysis of expression of 30 genes after treatment of NB cell lines with 5-Aza-dC or TSA

	**5-Aza-dC treatment**				**TSA treatment**			
**Genes**	**SK-N-AS**	**SK-N-BE(2)**	**IMR-32**	**SH-SY5Y**	**SK-N-AS**	**SK-N-BE(2)**	**IMR-32**	**SH-SY5Y**
*VAMP3*								
*PER3*						+		
*UTS2*		UD	UD			UD	UD	
*TNFRSF9*			UD	+	+	+	UD	+
*PARK7*								
*ERRFI1*					+	+	+	+
*RERE*							+	
*DKFZ566*							+	
*ENO1*								
*CA6*	UD	UD	UD	UD	UD	UD	UD	UD
*SLC2A5*	UD	UD	UD	+	+	UD	UD	
*GPR157*	+						+	
*H6PD*								
*SSB1*						+		
*MGC4399*								
*PIK3CD*	+	+			+	+	+	+
*CLSTN1*								
*ICAT*						+		
*LZIC*								
*NMNAT1*								
*RBP7*	+	+	ND		+	+	+	+
*UBE4B*								
*KIF1B*								
*PGD*								
*APITD1*								
*CORT*								
*DFFA*						+		
*PEX14*								
*CASZ1*			+	+	+	+	+	+
*TARDBP*								

UD=undetermined, gene transcripts not detected in the real-time PCR amplification.

ND=not determined.

Genes showing consistant upregulation after treatment are high-lighted in yellow.

**Table 3:** DNA variations detected in the study

**Gene**	**Gene position**	**Patient**	**NB Stage**	**1p-del**	**Outcome**	**Base change**	**Affected cases**	**Protein**	**Normal tissue from the patient**	**Healthy controls**
*Variations*										
*PIK3CD*	Exon 5	24R3	4	Pos	NED	448G>A	Heterozygous G/A	A150T	G/G	0/119
		24R3	4	Pos	NED	469C>A	Heterozygous C/A	L157M	C/C	0/119
		24R3	4	Pos	NED	562C>T	Heterozygous C/T	R188W	C/C	0/119
	Exon 16	19R6	1	Pos	DOD	1965G>A	Hemizygous A/−	M655I	G/A	0/113
	Intron 19	24R3	4	Pos	NED	IVS19+18C>T	Hemizygous T/−		C/C	0/112
	Exon 21	19R6	1	Pos	DOD	2661T>C	Hemizygous C/−	Y887Y	T/C	0/114
*ERRFI1*	Exon 4	25R9	2	Neg	NED	1028A>G	Heterozygous A/G	N343S	A/G	0/111
	Exon 4	24R3	4	Pos	NED	1186C>A	Heterozygous C/A	L396L	C/C	0/111
										
*PIK3CD*	Exon 8	18E4	4	Pos	DOD	935C>G	Homozygous G/G	S312S		6/112
		18E9	4	Pos	DOD	935C>G	Heterozygous C/G			
	Intron 7	23R7	4	Pos	DOD	IVS7-9G>C	Heterozygous G/C			2/112
		13E6	3	Pos	DOD	IVS7-9G>C	Homozygous C/C			
		15R3	4	Pos	DOD	IVS7-9G>C	Heterozygous G/C		G/C	
	Exon 18	10R2	4	Pos	DOD	2319C>T	Heterozygous C/T	S773G	C/T	1/112
		11R9	4	Pos	DOD	2319C>T	Heterozygous C/T		C/T	
*ERRFI1*	Exon 4, 3'UTR	11E2	4	Neg	DOD	1718A>G	Heterozygous A/G			4/102
	Exon 4, 3'UTR	12R6	4	Pos	DOD	1924A>G	Heterozygous A/G		A/G	4/117
*CASZ1*	Exon 2	14R9	2B	Pos	NED	1-309G>A	Heterozygous G/A			1/89
	Exon 8	17R4	4	Pos	DOD	1527G>A	Heterozygous G/A	K509K		2/112

1p-del=1p-deletion; Pos=positive; Neg=negative; NED=no evidence of disease; DOD=dead of disease.
